# Blood Plasma Metabolome Profiling at Different Stages of Renal Cell Carcinoma

**DOI:** 10.3390/cancers15010140

**Published:** 2022-12-26

**Authors:** Dmitry L. Maslov, Oxana P. Trifonova, Steven Lichtenberg, Elena E. Balashova, Zaman Z. Mamedli, Aleksandr A. Alferov, Ivan S. Stilidi, Petr G. Lokhov, Nikolay E. Kushlinskii, Alexander I. Archakov

**Affiliations:** 1 Institute of Biomedical Chemistry, 10 Building 8, Pogodinskaya Street, 119121 Moscow, Russia; 2 Metabometrics Inc., 651 N Broad Street, Suite 205 #1370, Middletown, DE 19709, USA; 3 N.N. Blokhin National Medical Research Center of Oncology of the Ministry of Health of Russia, 115478 Moscow, Russia

**Keywords:** renal cell carcinoma, metabolite profiling, biomarkers, direct mass spectrometry, blood plasma

## Abstract

**Simple Summary:**

Renal cell carcinoma (RCC) is one of the most common cancer types. However, the lack of clinical symptoms and validated biomarkers for early stage RCC prevent timely disease diagnosis. The study was focused on revealing potential low molecular biomarkers for early-stage RCC. The untargeted direct injection mass spectrometry-based metabolite profiling of blood plasma samples from non-cancer volunteers (control) and RCC patients (early stages of clear cell RCC (ccRCC), papillary RCC (pRCC), chromophobe RCC (chrRCC), and advanced stages of ccRCC) was performed. A set of metabolites with diagnostic power for the early stages of ccRCC was detected.

**Abstract:**

Early diagnostics significantly improves the survival of patients with renal cell carcinoma (RCC), which is the prevailing type of adult kidney cancer. However, the absence of clinically obvious symptoms and effective screening strategies at the early stages result to disease progression and survival rate reducing. The study was focused on revealing of potential low molecular biomarkers for early-stage RCC. The untargeted direct injection mass spectrometry-based metabolite profiling of blood plasma samples from 51 non-cancer volunteers (control) and 78 patients with different RCC subtypes and stages (early stages of clear cell RCC (ccRCC), papillary RCC (pRCC), chromophobe RCC (chrRCC) and advanced stages of ccRCC) was performed. Comparative analysis of the blood plasma metabolites between the control and cancer groups provided the detection of metabolites associated with different tumor stages. The designed model based on the revealed metabolites demonstrated high diagnostic power and accuracy. Overall, using the metabolomics approach the study revealed the metabolites demonstrating a high value for design of plasma-based test to improve early ccRCC diagnosis.

## 1. Introduction

Cancer along with cardiovascular diseases is the most common cause of death worldwide [1]. Renal cell carcinoma (RCC), also known as renal adenocarcinoma or hypernephroma, is one of the most common types of cancers and is diagnosed in more than 80% of adult kidney cancers. Three main histological subtypes of RCC include clear cell RCC (ccRCC), papillary RCC (pRCC), and chromophobe RCC (chrRCC), observing at 75–85%, 10–15%, and 5–10%, respectively [2,3,4]. The effectiveness of the disease treatment depends on how early it was diagnosed. However, an absence of obvious clinical symptoms and non-effective screening strategies especially at early stages of the disease lead to unnoticeable progression of the tumor and significant reduction in patient survival rate [5,6]. Wide application of modern effective methods of diagnostics (the fine needle biopsy, computed tomography scan (CT scan), magnetic resonance imaging (MRI), etc.) is limited by the high cost of the specialized equipment and qualification requirements to staff [7]. Therefore, the development of new effective, at the same time relatively simple and cheaper methods for kidney cancer biomarker-based laboratory diagnostics is very important. 

In the last decades, the use of different modern “-omics” sciences for revealing cancer-specific alterations at the genome, proteome, and metabolome levels has attracted great interest [8]. Metabolomics is one of the highly promising “-omics” that comprehensively analyses low-molecular-weight (typically less than 1000 Da) molecules (metabolites) in biological systems [9,10,11]. Metabolites being the substances, intermediates, and products of various biochemical reactions are the best reflection of the physiological and pathological processes, which take place in a human organism [12]. Significant metabolic alterations are one of a hallmark of many human disorders (age-related diseases, neurodegenerative disorders, autoimmune, inflammatory diseases, etc., including different cancer types) [13,14,15,16]. Numerous studies demonstrated the ability of metabolomic profiling-based approaches to provide a diagnostics of many diseases, prediction an effectiveness of proposed therapeutic strategies, and monitoring of selected treatment [16,17,18,19].

The untargeted direct injection mass spectrometry (DIMS)-based metabolome profiling of blood plasma samples from non-cancer volunteers (control) and patients with different RCC subtypes and the tumor stages (early stages of clear cell RCC (ccRCC), papillary RCC (pRCC), chromophobe RCC (chrRCC) and advanced stages of ccRCC) was performed to reveal metabolites associated with tumor progression.

## 2. Materials and Methods

### 2.1. Subject Collection

The venous blood samples were collected from 129 participants, including 51 non-cancer volunteers (the subjects without a tumor in the past) and 78 patients with different RCC stages (Table 1) at the Federal State Budgetary Institution “N.N. Blokhin National Medical Research Center of Oncology” of the Ministry of Health of Russia (Moscow, Russia) from September 2017 to September 2019. 

The twenty-five lung cancer plasma samples were used as an additional control to assess the specificity of the revealed set of the most promising metabolites chosen for the ccRCC diagnostic model. These samples were used in the previously published study [20] and are stored in the laboratory biobank at −80 °C.

The presence of kidney cancer in patients was confirmed by clinical, laboratory, and morphological research methods. Histological variants of renal cell carcinoma were identified according to the international WHO classification [21]. The clinical stages of RCC were assigned according to the 2009 Classification of Malignant Tumors (TNM), which includes tumor size, invasion into the inferior vena cava, capsule invasion, involvement of the adrenal gland and lymph nodes, and the presence of distant metastases. The RCC patients with severe diseases (diabetes and others, including other cancer types) were excluded from the study. All procedures performed in the investigations involving human subjects were in accordance with the ethical standards of the institutional and national research committee and with the 1964 Helsinki declaration and its later amendments or comparable ethical standards. 

### 2.2. Study Design

The experiment was designed as case–control study to determine alterations in plasma metabolite composition associated with the RCC progression. For this purpose, the recruited subjects with RCC were divided into several groups according to the kidney cancer type, and degree of tumor progression. The design of the comparative analysis (summary list of the compared group pairs) is shown in Table 2.

### 2.3. Sample Preparation

Blood samples were collected in the morning after overnight fasting into EDTA Vacutainer plasma tubes (BD, Franklin Lakes, NJ, USA) and cooled down at 4 °C immediately. Blood plasma was separated by centrifugation according to the manufacturer’s instruction (4000 rpm for 10 min at 4 °C), transferred into a clean 2 mL Eppendorf, and immediately stored at −80 °C until analysis. For analysis, the frozen plasma samples were thawed on ice, and an aliquot (10 µL) was mixed with 80 µL pre-cooled methanol (J.T. Baker, Gliwice, Poland) and 10 µL water (Sigma-Aldrich, St. Louis, MO, USA). The mixture was incubated for 10 min (on ice with periodical shaking) and centrifuged (13,000× *g*, 4 °C, 15 min). The supernatant was transferred to a clean 2 mL Eppendorf and 10 µL of the supernatant was mixed with fifty volumes of methanol containing 0.1% formic acid (Fluka, Munich, Germany). As an internal standard, 0.4 µL (5 mg/L) of losartan solution was added. The resulting solutions were analyzed by direct infusion mass spectrometry. 

### 2.4. Metabolite Profiling

A hybrid quadrupole time-of-flight mass spectrometer (maXis Impact, Bruker Daltonics, Billerica, MA, USA) equipped with electrospray ionization (ESI) was applied for the analysis of the metabolomic composition of samples. Full scan data acquisition was performed in positive ion mode over the range of mass-to-charge ratio (*m/z*) from 50 to 1000 with a mass accuracy of 1–3 parts per million (ppm). The mass spectrometer was calibrated daily by applying external calibration standard ES Tuning Mix (Agilent Technologies, Santa Clara, CA, USA). The samples were injected into the ESI source by using of a precision glass syringe (Hamilton Bonaduz AG, Bonaduz, Switzerland) and a syringe pump (KD Scientific, Holliston, MA, USA) with the flow rate of 180 µL/h. MS analysis of the samples (kidney cancer patients, controls, and blank samples) was carried out in a randomized order. Mass spectra were recorded by DataAnalysis software (version 4.1, Bruker Daltonics) to summarize signals for 1 min. Three technical replicates per sample were performed.

### 2.5. Mass Spectrum Processing

The MS raw data were processed by DataAnalysis software (version 4.1, Bruker Daltonics, Bremen, Germany). The following parameters were used for mass peak detection: peak width, 3; signal–to–noise ratio, 2; and relative and absolute threshold intensity, 0.05% and 100, respectively. Normalization of MS peak intensities was performed as described previously [20]. Alignment of the *m/z* values of the mass peaks to the different mass spectra was performed as described previously [22].

### 2.6. Statistical Analysis

After preprocessing, the data were subjected to statistical analysis to identify discriminated metabolite mass peaks (variables). Orthogonal partial least squares discriminant analysis (OPLS-DA) was performed using the MetaboAnalyst 5.0 (www.metaboanalyst.ca, accessed on 14 June 2022), a free online software for metabolomics data analysis [23]. R^2^Y and Q^2^ parameters were used to assess the quality of the OPLS-DA models. The fitness of the computed models was evaluated through one hundred permutation validations (*p*-value ≤ 0.05 was considered as statistically significant). Variable importance for the projection (VIP) values was applied to rank the variables according to their contribution to the discrimination of the compared experimental groups. Univariate statistical analysis (pairwise Mann–Whitney *U* test), implemented in Statistica software 10.0 (StatSoft Inc., Tulsa, Oklahoma, USA), was utilized to evaluate the significance of the variables. Implemented in MetaboAnalyst 5.0 unpaired Wilcoxon rank-sum test and the false discovery rate (FDR) with threshold *p* < 0.05 were applied for reconfirmation and correction of the results. The final selection of the most influent variables was based on the following conditions: VIP value obtained from OPLS-DA > 1.0, and *p*-value < 0.05. 

Further, the optimal combination of metabolites for effective discrimination of RCC patients and non-cancer patients was revealed. The subjects were randomly divided into two groups. The first group (independent test set) included 1/3 of the early stage of ccRCC subjects (six males and seven females) and non-cancer controls (seven males and eight females). This group was not included in the model building process. The second group (training and cross validation (CV) sets for the model) included the remaining 2/3 of the early stage ccRCC subjects (12 males and 14 females) and non-cancer controls (16 males and 20 females). The Biomarker analysis tool (MetaboAnalyst 5.0) was used to construct diagnostic models, and their efficiency was estimated using the area under the ROC curve (AUC) calculated by Monte-Carlo cross validation (MCCV) algorithm. The diagnostic models based on metabolite combinations were ranked according to predictive power by Random Forest algorithm (classification and feature ranking). One hundred-times repeated 3-fold CV (100-times) was used for the evaluation of the reliability of the models. 

To avoid overfitting, the estimation of the most optimal model was performed by using the independent test set (these data were not used for the machine learning) without class labels. For this, the Tester module (MetaboAnalyst 5.0) was used. The class labels of the independent test set predicted by the Tester module were compared to actual labels, and the predictive effectiveness was evaluated using AUC, sensitivity, specificity, Nagelkerke R^2^ (SPSS software (ver. 10.0.7, IBM, Chicago, Illinois, USA); MedCalc software, https://www.medcalc.org/calc/diagnostic_test.php, accessed on 14 December 2022) and Brier score (using formula from [24]).

To exclude the possible influence of imbalance of the data set (51 samples in control group vs. 39 samples in early stage ccRCC group) on correctness of model performance, the data set was balanced by random removing of control samples till the equal samples size (down-sizing approach) [25]. 

The set of the most promising metabolites chosen for the diagnostic model was also tested for discrimination between the early stage ccRCC and advanced stage ccRCC, as well as control and early-stage lung cancer groups. For this, the above-described approaches with independent test sets and balanced data sets were applied.

### 2.7. Metabolite Annotation

Annotation of the significantly different variables with a clear isotope pattern was based on matching the accurate mass (*m/z*) of analyte of interest and its isotopic distribution against data of authentic compounds deposited in Human Metabolome Database (HMDB) (http://www.hmbd.ca, accessed on 14 June 2022) [26] and METLIN (http://metlin.scripps.edu, accessed on 14 June 2022) [27]. A tolerance range of molecular weights for the mass-based search was 0.01 Da. Isotope Pattern Calculator (Bruker Daltonics, Germany) was used for the generation of a theoretical isotope pattern. Metabolite annotation based on matching of at least two independent and orthogonal properties (accurate mass and isotopic distribution) is satisfied to the second level of identification confidence (putatively annotated compounds), according to the Metabolomics Standards Initiative (MSI) guidelines [28]. The structure of acyl chains of phospholipids was not elucidated. Therefore, the identification of phospholipids corresponds to level 3 (putatively annotated compound classes) according to the MSI guidelines. The tandem mass spectrometry (MS/MS) approach was applied for the validation of the identification of selected metabolites. Experimental MS/MS fragmentation patterns of ions of interest (obtained at different collision energies (from 10 to 40 eV) in positive ionization mode) were compared against MS/MS fragmentation products data derived in the metabolite databases (HMDB and METLIN).

### 2.8. Pathway Analysis

To reveal metabolic pathways associated with RCC development, metabolic pathway analysis (MetPA) was performed using the MetaboAnalyst 5.0. Two popular algorithms of biological pathways estimations are combined in the MetPA module: metabolite set enrichment analysis (MSEA) and topology analysis (TA) [29]. Thus, the reveal of the statistically significant differentially abundant pathways is based on two independent parameters: *p*-value computed by MSEA and impact value calculated by the TA. KEGG (Kyoto Encyclopedia of Genes and Genomes) human metabolic network was used in the analysis (https://www.genome.jp/kegg/, accessed on 14 June 2022). The impact values over 0.1 and the *p* ≤ 0.05 were taken as the thresholds [30].

## 3. Results

### 3.1. Mass Spectrometry Analysis

The metabolomic profiling of plasma samples using direct injection mass spectrometry (DIMS) was performed. Over 9000 mass spectrometry peaks with the mass-to-charge ratio (*m/z*) of 50–1000 were detected. The preprocessing procedure eliminated mass peaks from the subsequent analysis if they were missed in 25% or more samples in every group. 

### 3.2. Statistical Analysis and Metabolite Annotation

**Figure 1:**
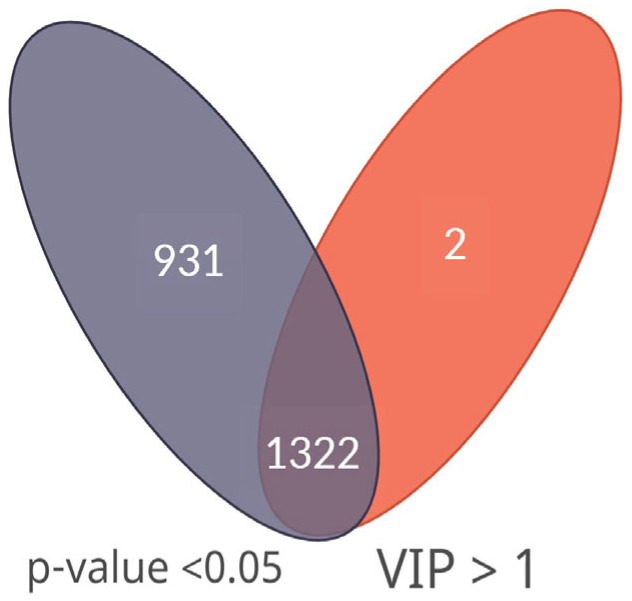
Selection of the most discriminatory *m/z* peaks to further analysis. The Venn diagram demonstrates the number of detected peaks common for two statistical approaches (Variable importance for the Projection (VIP) and Wilcoxon rank-sum test using False Discovery Rate (FDR) corrected *p*-values).

The selection of differential mass spectrometry peaks is particularly critical as it may significantly influence on the final biological interpretation. The combined application of various statistical techniques is widely recommended approach for metabolomics analysis [31]. Using of multiple statistical methods allows to avoid mistakes related to applying one method only and provides an effective selection of the most important discriminatory *m/z* peaks. The obtained data were processed by multivariate and univariate statistical analysis aimed to identify peaks with most meaningful contribution to group differences. For multivariate statistical analysis, OPLS-DA was used. The constructed OPLS-DA models demonstrated segregation between the control and all cancer groups (Supplementary Figure S1). Parameters of OPLS-DA models (R^2^Y, Q^2^, and *p*-value) are summarized in Supplementary Table S1. The VIP approach was applied to select differential peaks in the OPLS-DA models (peaks with VIP value >1.0 were considered as the significant contributors). For univariate analysis, the statistical significance of peaks intensity difference between the experimental groups was evaluated by *p*-value (*p* below 0.05 was considered as statistically significant). The combined application of two statistical techniques provided detection of the most important discriminatory *m/z* peaks. The peaks characterized by VIP >1.0 in multivariate statistical analysis and *p*-value <0.05 in univariate analysis were selected to further analysis (Figure 1).

Among the discriminatory *m/z* peaks 67 metabolites were putatively annotated (Supplementary Table S2). The variation in the levels of some annotated metabolites between the groups (controls and RCC patients at different stages) is illustrated in Supplementary Figure S2. MS/MS confirmed the identification of twelve metabolites (Supplementary Table S3). These metabolites were lipids, amino acids, and carbohydrates. Most of the annotated metabolites were down-regulated in cancer patients at all stages. An alteration of plasma level for such metabolites as oxoproline, taurine, phenylalanine, tyrosine, citrulline, and some PCs exhibited a clear correlation with tumor progression (Supplementary Figure S2). At the same time, results of multivariate statistical analysis failed to separate different RCC subtypes at early stages. 

### 3.3. Pathways Associated with ccRCC Progression

**Figure 2:**
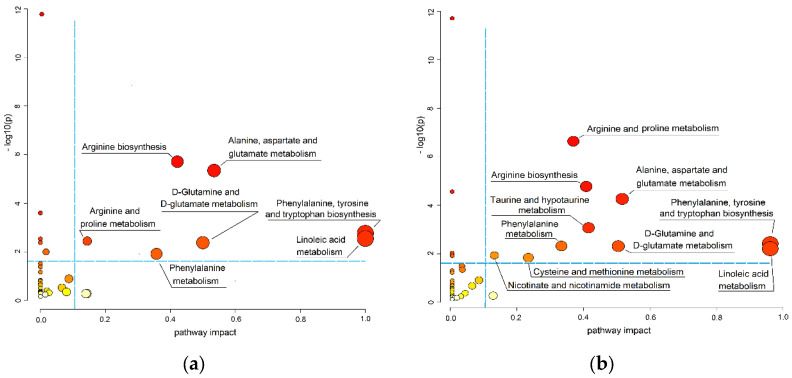
Pathway analysis (MetPA) of metabolites associated with different stages of ccRCC (KEGG database was used): (**a**) Metabolic pathways associated with I–II stages ccRCC; (**b**) Metabolic pathways associated with III–IV stages ccRCC. The *y*-axis—the negative natural logarithmic value of the original *p*-value, which indicates the pathway enrichment analysis. The *x*-axis indicates the impact values representing pathway topology analysis. The identified pathways are displayed as circles. The color of the circles is correlated with negative log(p) values (dark red indicates a highly significant, light yellow—insignificant), the size of the circles indicates the pathway impact value (an increase in the size is associated with the growth of impact value). The negative log(p) values lower than 1.5 (*p*-values lower than 0.05) and impact values 0.1 were taken as the thresholds.

In the next step, the determination of the link between alterations of metabolomics composition and biological context was performed. Based on the list of putatively annotated metabolites (Supplementary Table S2), the MetPA revealed the metabolic pathways potentially associated with the pathogenesis of ccRCC. The information about the pathways dysregulated at various stages of the disease is summarized in Table 3 and visualized in Figure 2.

It was found that the revealed pathways are predominantly related to amino acid metabolism. The perturbed metabolic pathways revealed in the patients with early stages of ccRCC are associated with glutamate, glutamine, arginine, proline, phenylalanine, tyrosine, tryptophan, and linoleate metabolism (Table 3, Figure 2a). Along with biological pathways that were disordered in the early stages, the taurine, hypotaurine, cysteine, methionine, and nicotinamide metabolism are dysregulated at the late stages of the disorder ((Table 3, Figure 2b). An increase in significant hits numbers (means the number of significantly changed metabolites involved in the particular pathway) accompanied with disease progression can be noted (Table 3). This fact shows an enhancement of dysregulation of the biological pathways related to disease progression. Should be noted that the same dysregulated pathways were detected for both ccRCC and pRCC/chrRCC.

### 3.4. Predictive Power of the Selected Metabolites for Early Stages ccRCC

**Figure 3:**
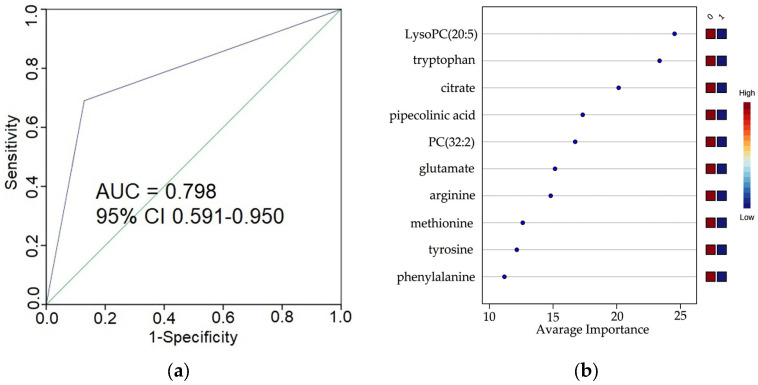
The estimation of the constructed diagnostic model for early stage ccRCC: (**a**). ROC curve and AUC of diagnostic model (generated using 10 metabolites) calculated by using the independent test set. SPSS was used to build the plot; (**b**). Metabolites ranked by their contributions to classification accuracy. The Random Forest algorithm was used. The colored boxes on the right indicate the intensity of the corresponding metabolite *m/z* peak in the groups (0—control, 1—ccRCC early-stage patients).

Further, the diagnostics efficiency of the annotated metabolites was evaluated by calculation of AUC. The metabolites characterized by AUC>0.7 were selected. To obtain higher diagnostic performance, the different combinations were constructed from the top 14 metabolites. The model constructed using the 10 metabolites (citrate, glutamate, arginine, tyrosine, phenylalanine, methionine, tryptophan, pipecolinic acid, lysoPC (20:5), and PC (32:2)) demonstrated the highest discrimination efficiency between the control and ccRCC early stages (Supplementary Figure S3a,b). On the next stage, the model performance was estimated by the independent test set. The following values for the independent test sets were obtained: AUC—0.80 (95% CI: 0.59–0.95); sensitivity—0.79 (95% CI: 0.74–0.84); specificity—0.82 (95% CI: 0.76–0.88)**;** Nagelkerke R^2^—0.475; -2 Log likelihood—81.04; Cox and Snell R^2^—0.32 (Figure 3). A Brier score of 0.127 displays a good discrimination between samples of early stage ccRCC patients and controls. The overfitting-corrected calibration plot is shown in Supplementary Figure S3c. The performance of the model, as estimated on the independent test set, can be considered relatively high. 

MS/MS analysis confirmed the identification of five metabolites chosen for the construction of the diagnostic model (Supplementary Table S3). The metabolites included in the diagnostic model are associated with the revealed biological pathways involved in kidney cancer pathogenesis (Table 4). 

The detailed study of the model for prediction of early stage ccRCC does not demonstrate sex-related difference of the model performance (Supplementary Figure S4, Table S4).

Moreover, there was no significant change in the AUC values of the model for prediction of early stage ccRCC after correction of the imbalance of data set. Thus, the obtained results indicate that the difference between the number of control and early stage ccRCC samples in this study does not affect the performance of the model. 

### 3.5. Evaluation of the Diagnostic Model for the ccRCC Advanced Stages

In addition, the ability of the early stage ccRCC diagnostic model to distinguish early (I–II stages) and advanced (III–IV stages) stages of ccRCC was studied (Supplementary Figure S5). The following values were obtained using independent test set: AUC—0.70 (95% CI: 0.60–0.81), sensitivity –0.82 (95% CI: 0.75–0.88), and specificity—0.74 (95% CI: 0.62–0.83). These results indicates that the diagnostic model has poor performance for discrimination of different stages of ccRCC (early vs. advanced stages).

### 3.6. Evaluation of Specificity of RCC Diagnostic Model on Lung Cancer Samples

Lung cancer plasma samples (early stage) were used as an additional control to assess the specificity of the early stage ccRCC diagnostic model (Supplementary Figure S6). The following values were obtained using independent test set: AUC– 0.60 (95% CI: 0.46–0.74), sensitivity– 0.61 (95% CI: 0.48–0.73), and specificity—0.69 (95% CI: 0.49–0.89). The results demonstrates that the early stage ccRCC diagnostic model has poor performance for the detection of lung cancer.

Due to a lack of samples for the independent test set, the efficiency of the model for diagnosing early stage pRCC and chRCC was not validated.

## 4. Discussion

The metabolome is a final level of organization of biological systems directly related to global biochemical phenotype [10]. Metabolomics with its ability to detect numerous sets of metabolites, i.e., metabolome, allows precisely differentiate cases from the controls based on multivariate characteristics—molecular assembles, metabolic fingerprints, signatures, etc. These multivariate characteristics are expected to describe global biochemical aberrations that reflect variances in state of wellness and may describe diseases and their progression more accurately. Thus, metabolomics is able to greatly aid in the differential diagnosis. Taking into account that laboratory diagnostics of early stages of RCC is still challenging, the application of metabolomics analysis of blood from RCC patients in this study was actual.

Despite several limitations, the DIMS is widely used in modern metabolomics investigations [32,33,34]. Due to technological advancements (application of modern high-resolution mass spectrometers, data processing procedures, etc.) the approach enables produce the high-quality metabolomics data with high sensitivity, accurate mass measurements, and wide dynamic range of detection [35]. In addition DIMS approach is characterized by high sample throughput (that especially actual for large-scale studies), high reproducibility and relatively low consumables cost per analyzed sample [35,36].

A coupling of statistical analysis of metabolomic data and specialized bioinformatic software enabled to detect the metabolites involved in discrimination between the RCC patients and controls (non–cancer volunteers) and to reveal the biological pathways linked to kidney cancer progression. 

Most of the annotated discriminatory metabolites and relevant biological pathways were directly associated with amino acid and lipids metabolism. The selected amino acids and their derivatives are involved in processes that are required for cellular growth and proliferation. These amino acids are alternative energy sources [37,38], the source of protein synthesis [39] and important components of complex protection system from reactive oxygen species that accumulated during the active proliferation of cells [40]. Moreover they are involved in regulation of DNA synthesis [41], anabolic and proliferative activity [42], etc. The lipids play key roles in numerous cellular processes linked to cellular growth and proliferation. They are involved in membrane formation, regulation of signaling processes, and can be used as the source for enhancement of the beta-oxidation process [43,44]. 

The findings of this study support and supplement the findings of previous RCC related metabolomics studies focusing on the metabolite profiling of kidney tissues [45,46] and various biofluids (plasma [47,48], serum [49,50], and urine [51,52,53]). The detected in this study association between alteration of some amino acids level (such as leucine, valine, and tryptophan) and kidney cancer progression was supported by multiple previous studies [42,54]. The similar alterations of some other revealed discriminatory amino acids such as arginine, tyrosine, phenylalanine, and methionine were observed in plasma-based metabolomics studies of different cancer types (such as lung, gastric, colorectal, breast, prostate, pancreatic, esophageal, and endometrial cancers) [42,55,56]. The link between disturbance of some metabolomic pathways revealed in the study (such as tryptophan metabolism, arachidonic acid metabolism, phospholipids metabolism, and linoleic acid metabolism) and disease progression was also supported by previous observations in patients with different stages of RCC [48,50,57]. The perturbations of some other metabolomic pathways (such as arginine-related pathways and glutamine associated pathways) were found in other cancer types (such as collateral, prostate, lung, and liver cancers) [58,59,60]. Probably, the observed metabolomics changes common for different cancer types (including the RCC subtypes) reflect the dysregulation similarity of many biological processes. Most likely, the cause of the observed effects is the reprogramming of cellular metabolism leading to intensify growth and proliferation processes to provide a survival of neoplastic cells [61]. The enhancement in the amino acid and lipids demand and improvement cellular uptake efficiency by tumor cells may be one of the most likely causes of the observed decline of the plasma metabolites level in cancer patients [62]. However, the degree of perturbation of the biological pathways is significantly different in various cancer types that provide cancer types classifying and background for cancer-specific diagnostic signatures [63].

Plasma and serum samples were used in numerous previously published metabolomics studies aimed the segregation of RCC subtypes and non-cancer control groups [64,65]. The benefit of the study is focusing on detection by MS-based approach of the plasma differential metabolites distinguishing control from cancer cases with different stages of ccRCC and identification of relevant biological pathways. The discovery of alterations in metabolomics composition and relevant pathways associated with various kidney cancer stages can provide deeper insight into the tumor biology. The knowledge may help to understand the underlying mechanisms of kidney cancer initiation and progression on the molecular level. An ability of untargeted metabolomics to detect dysregulated metabolic pathways in RCC patients offers an opportunity for a broader and more integrated monitoring of patient’s status. It can help in choice of most effective therapeutic strategy. In addition, the revealing of the dysregulated metabolomic pathways associated with the cancer initiation and progression can provide a design of new antitumor treatment and developing individualized therapeutic strategies based on individual disturbances of the biological pathways. 

Despite the numerous RCC focused metabolomics studies using the well-established analytical platforms (NMR spectroscopy, LC-MS, and GS-MS) the application of the DIMS-based approach allowed to reveal new potential diagnostic pattern for ccRCC early diagnosis not previously described. The pattern is characterized by high value of sensitivity and specificity and demonstrates good predictive accuracy. The contribution of most of the pattern’s metabolites to the pathogenesis of ccRCC was supported by multiple previous studies [47,48]. This fact can be considered as one of the ways of confirmation of pattern reliability. Due to metabolic alterations may be common for different cancer types [66,67], it is better to additionally test the revealed ccRCC diagnostic pattern on another cancer type. The poor diagnostic power of the ccRCC diagnostic pattern was demonstrated on lung cancer samples. This suggests a cancer-type specificity of the identified diagnostic pattern. Moreover, should be noted that the founded in the study ccRCC diagnostic pattern does not allow different stages of disorder to be reliably distinguished. Most likely, this indicates that the diagnostic pattern is based on ccRCC-specific metabolic alterations that are actual for all stages.

The ccRCC diagnostic pattern testing showed a lack of the gender-related differences of this pattern performance. However, this fact does not contradict the results of other published studies where gender-associated diagnostic patterns were demonstrated. Because the design of gender-specific diagnostic patterns was not the goal of this study, the discriminatory metabolites were not selected based on sex, and a gender-nonspecific pattern was generated.

Some studies demonstrated the very high diagnostic efficiency of RCC metabolomic patterns (AUC > 0.90–0.95) [49,68,69]. However, the detailed analysis of these studies shows that the presented results can be over-optimistic. The reason is the incorrect data set selection for the model construction and efficiency estimation. This mistake is widespread distributed in biomarker studies using the machine learning approach [70]. The using of the same samples in training and test data sets and/or lack of the validation of the model’s accuracy by the independent data set leads to overstatement model efficiency [70,71,72]. This fact is confirmed by the results of the studies where independent test set was applied and AUC for efficiency of constructed model was adjusted to the lower value [73,74].

Several limitations of this study should be considered. The identification of the detected potential candidates is corresponded to levels 2 and 3 according to the Metabolomics Standards Initiative (MSI) guidelines. Such identification meets the requirements of biological research. However, level 1 is needed for further implementation of the identified metabolomic patterns in clinical practice. For this purpose, in future, the most robust identification of the detected candidate biomarkers should be performed with isotope-labeled standards. In addition, the investigation by means of cell lines can be performed in the future to provide more accurate information about contribution of the selected metabolites to RCC progression. Another limitation of the current study is the small sample sizes. In future, studies with larger sample sizes and different cancer types as an additional control are needed for more robust validation of the diagnostic value of the detected metabolomic pattern.

In prospect, the diagnostic pattern can be used to design new effective approach for the diagnostics of early stages of ccRCC. The detection of early stages of cancer is one of the actual goals of modern clinical diagnostics as a key factor of treatment effectiveness. We believe that in future, the accumulation of the knowledge about RCC biomarkers revealed in various studies can provide a design of early diagnostic tool, which would be sensitive and specific enough for early cancer prediction. The discovery of such diagnostic tools can provide a timely treatment that facilitates improvement of patients’ outcomes and significant increase in the overall survival of patients.

Thus, the analysis of the obtained data confirmed that the metabolomics study of blood plasma globally reflects the biochemical phenotype of the organism, sensitive to RCC-specific aberrations. This was a rigorous scientific basis to propose a multivariate RCC diagnosis based on the most promising set of metabolites resulting in high diagnostic accuracy for early stages.

## 5. Conclusions

Early diagnosis of RCC is difficult; therefore, the use of modern scientific research tools, such as metabolomic analysis, can overcome the existing problems. Using a metabolomic blood analysis based on direct mass spectrometry it was possible to panoramically identify RCC-specific changes and to propose a scientifically based model for the early diagnosis of the disease with high accuracy. 

## Figures and Tables

**Table 1:** Summary of the cohorts.

Cohort	Total Number	Age (years)	BMI (kg/m^2^)	Male		Female		Male/Female
				**Number**	**Age** **(years)**	**Number**	**Age** **(years)**	
Control	51	56.5 ± 7.7 ^1^	30.5 ± 2.7 ^1^	23	52.5 ± 6.9	28	59.1 ± 7.1	45%/55%
ccRCC (I–II stages)	39	60.0 ± 7.9	32.6 ± 3.4	18	57.1 ± 9.1	21	62.0 ± 6.5	46%/54%
ccRCC (III–IV stages)	22	58.3 ± 7.0	31.2 ± 2.8	17	57.5 ± 7.5	5	61.3 ± 4.1	77%/23%
pRCC and chrRCC (I–II stages)	12	58.2 ± 10.2	32.1 ± 2.6	4	57.3 ± 11.8	8	58.6 ± 9.4	33%/67%
Lung cancer ^2^ (I–II stages)	25	61.6 ± 4.2	30.3 ± 2.8	16	60.4 ± 3.5	9	62.3 ± 5.4	64%/36%

^1^ mean ± s.d.; ^2^ the lung cancer samples were used as additional control to evaluate the specificity of diagnostic pattern for ccRCC; ccRCC, clear cell renal cell carcinoma; pRCC, papillary renal cell carcinoma; chrRCC, chromophobe renal cell carcinoma.

**Table 2:** Groups of samples for comparative analysis.

Controls		Cancer Patients
non-cancer volunteers	vs	patients with ccRCC (I–II stages)
		patients with ccRCC (III–IV stages)
		patients with pRCC and chrRCC (I–II stages)

**Table 3:** Metabolic pathways altered at ccRCC.

№	Pathway Name ^1^	Total	Hits	*p*-Value	−log(*p*)	Impact
ccRCC and pRCC/chrRCC patients (early stages)						
1	Aminoacyl-tRNA biosynthesis	48	13	6.29 × 10^−14^	13.20	0.00
2	Arginine biosynthesiss ^2^	14	5	1.96 × 10^−6^	5.71	0.42
3	Alanine, aspartate, and glutamate metabolism ^2^	28	6	4.51 × 10^−6^	5.35	0.53
4	Valine, leucine, and isoleucine biosynthesis	8	3	2.49 × 10^−4^	3.60	0.00
5	Phenylalanine, tyrosine, and tryptophan biosynthesis ^2^	4	2	1.72 × 10^−3^	2.76	1.00
6	Linoleic acid metabolism ^2^	5	2	2.83 × 10^−3^	2.55	1.00
7	Biosynthesis of unsaturated fatty acids	36	4	2.94 × 10^−3^	2.53	0.00
8	Arginine and proline metabolism ^2^	38	4	3.60 × 10^−3^	2.44	0.14
9	Nitrogen metabolism	6	2	4.20 × 10^−3^	2.38	0.00
10	D-Glutamine and D-glutamate metabolism ^2^	6	2	4.20 × 10^−3^	2.38	0.50
11	Phenylalanine metabolism ^2^	10	2	1.21 × 10^−2^	1.92	0.36
12	Glyoxylate and dicarboxylate metabolism	32	3	1.67 × 10^−2^	1.78	0.03
13	Histidine metabolism	16	2	3.02 × 10^−2^	1.52	0.00
14	Lysine degradation	25	2	6.85 × 10^−2^	1.16	0.00
15	Glycerophospholipids metabolism	36	2	1.28 × 10^−1^	0.89	0.11
ccRCC patients (advanced stages)						
16	Aminoacyl-tRNA biosynthesis	48	14	1.29 × 10^−12^	11.89	0.00
17	Arginine and proline metabolism ^2^	38	9	1.88 × 10^−7^	6.73	0.38
18	Arginine biosynthesis ^2^	14	5	1.50 × 10^−5^	4.82	0.42
19	Valine, leucine, and isoleucine biosynthesis	8	4	2.47 × 10^−5^	4.61	0.00
20	Alanine, aspartate, and glutamate metabolism ^2^	28	6	4.98 × 10^−5^	4.30	0.53
21	Taurine and hypotaurine metabolism ^2^	8	3	8.16 × 10^−4^	3.09	0.43
22	Phenylalanine, tyrosine, and tryptophan biosynthesis ^2^	4	2	3.77 × 10^−3^	2.42	1.00
23	Linoleic acid metabolism ^2^	5	2	6.18 × 10^−3^	2.21	1.00
24	Nitrogen metabolism	6	2	9.12 × 10^−3^	2.04	0.00
25	D-Glutamine and D-glutamate metabolism ^2^	6	2	9.13 × 10^−3^	2.04	0.50
26	Biosynthesis of unsaturated fatty acids	36	4	1.23 × 10^−2^	1.91	0.00
27	Phenylalanine metabolism ^2^	10	2	2.56 × 10^−2^	1.59	0.36
28	Glutathione metabolism	28	3	3.33 × 10^−2^	1.47	0.03
29	Glyoxylate and dicarboxylate metabolism	32	3	4.47 × 10^−2^	1.35	0.03
30	Glycine, serine, and threonine metabolism	33	3	4.77 × 10^−2^	1.32	0.00
31	Cysteine and methionine metabolism ^2^	33	3	4.77 × 10^−2^	1.32	0.26
32	Nicotinate and nicotinamide metabolism ^2^	14	2	4.95 × 10^−2^	1.27	0.14
33	Histidine metabolism	16	2	6.20 × 10^−2^	1.21	0.00
34	Lysine degradation	25	2	1.34 × 10^−1^	0.87	0.00

^1^ pathways were sorted by their *p*-value. ^2^ pathways with *p*-values < 0.05 and impact values > 0.1 are marked by bold. Total—the number of all metabolites that are involved in the particular pathway; hits—the number of predicted metabolites from the dataset for the particular pathway; *p*-value—indicates the pathway enrichment analysis; −log(*p*)—the negative log_10_(*p*) values; impact—impact values representing pathway topology analysis.

**Table 4:** Biological pathways associated with the metabolites included in the diagnostic model.

Metabolites	Pathway Name ^1^
pipecolinic acid	arginine and proline metabolism
glutamate	alanine, aspartate, and glutamate metabolism
	arginine biosynthesis
	arginine and proline metabolism
	glutamine and glutamate metabolism
methionine	cysteine and methionine metabolism
arginine	arginine biosynthesis
	arginine and proline metabolism
tyrosine	phenylalanine, tyrosine, and tryptophanbiosynthesis
	phenylalanine metabolism
phenylalanine	phenylalanine, tyrosine, and tryptophanbiosynthesis
	phenylalanine metabolism
tryptophan	phenylalanine, tyrosine, and tryptophanbiosynthesis
citrate	alanine, aspartate, and glutamate metabolism

^1^ revealed biological pathways disturbed in the RCC patients.

## Data Availability

The data presented in this study are available on request from the corresponding author.

## Supplementary Materials

The following supporting information can be downloaded at: https://www.mdpi.com/article/10.3390/cancers15010140/s1, Figure S1: OPLS-DA score plots of metabolic profiles of blood plasma samples involved in the study; Figure S2: Box and whisker plots of the annotated metabolites between the controls and RCC patients at different stages. Figure S3: The selection of metabolites for the diagnostic model and its performance evaluation; Figure S4: The ability of the diagnostic model (generated using 10 metabolites) to discriminate the patients with early stage ccRCC according to their gender; Figure S5: Receiver operating characteristic (ROC) curve showing the ability of the diagnostic model (generated using 10 metabolites) to distinguish the early stage ccRCC samples from advanced stage ccRCC samples; Figure S6: Receiver operating characteristic (ROC) curve of the diagnostic model (generated using 10 metabolites) for lung cancer prediction; Table S1: Accuracy results of orthogonal partial least squares discriminant analysis models; Table S2: Putatively annotated differential metabolites; Table S3: Identification of differential metabolites by MS/MS fragmentation; Table S4: A discrimination ability of the model for prediction of early stage ccRCC.
