# Supplementary material for: Blood Plasma Metabolome Profiling at Different Stages of Renal Cell Carcinoma
Source: Cancers (Basel). 2022 Dec 26;15(1):140. doi: 10.3390/cancers15010140 (PMC9818272; doi:10.3390/cancers15010140)
Supplement: Supplementary file 1 [file cancers-15-00140-s001.zip › cancers-1971224-supplementary.pdf]

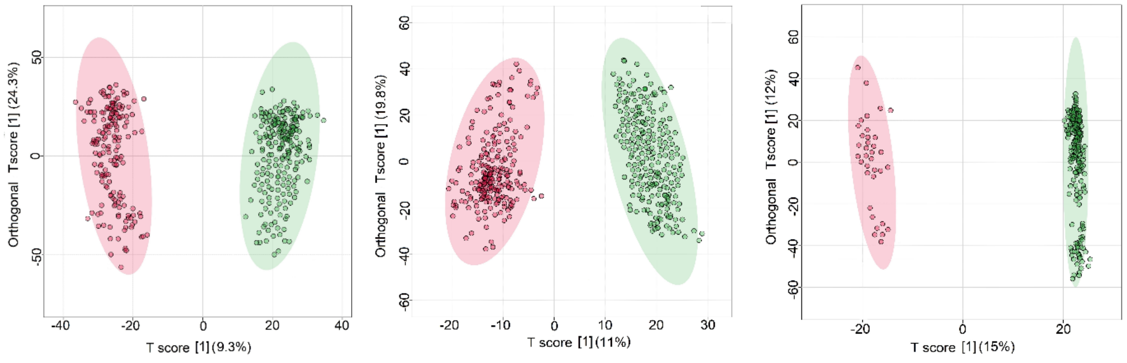

(a) **(b)** **(c)**

Figure S1. OPLS-DA score plots of metabolic profiles of blood plasma samples involved in the study. Plots demonstrate the distribution of ccRCC patient's samples (●) and samples of non-cancer volunteers (controls) (●)). (**a**) – control vs ccRCC patients (III-IV stages); (**b**) – control vs ccRCC patients (I-II stages); (**c**) – control vs pRCC and chrRCC patients (I-II stages). The detailed model validation parameters (R^2 and Q^2) for each statistical analysis are summarized in Table S1.

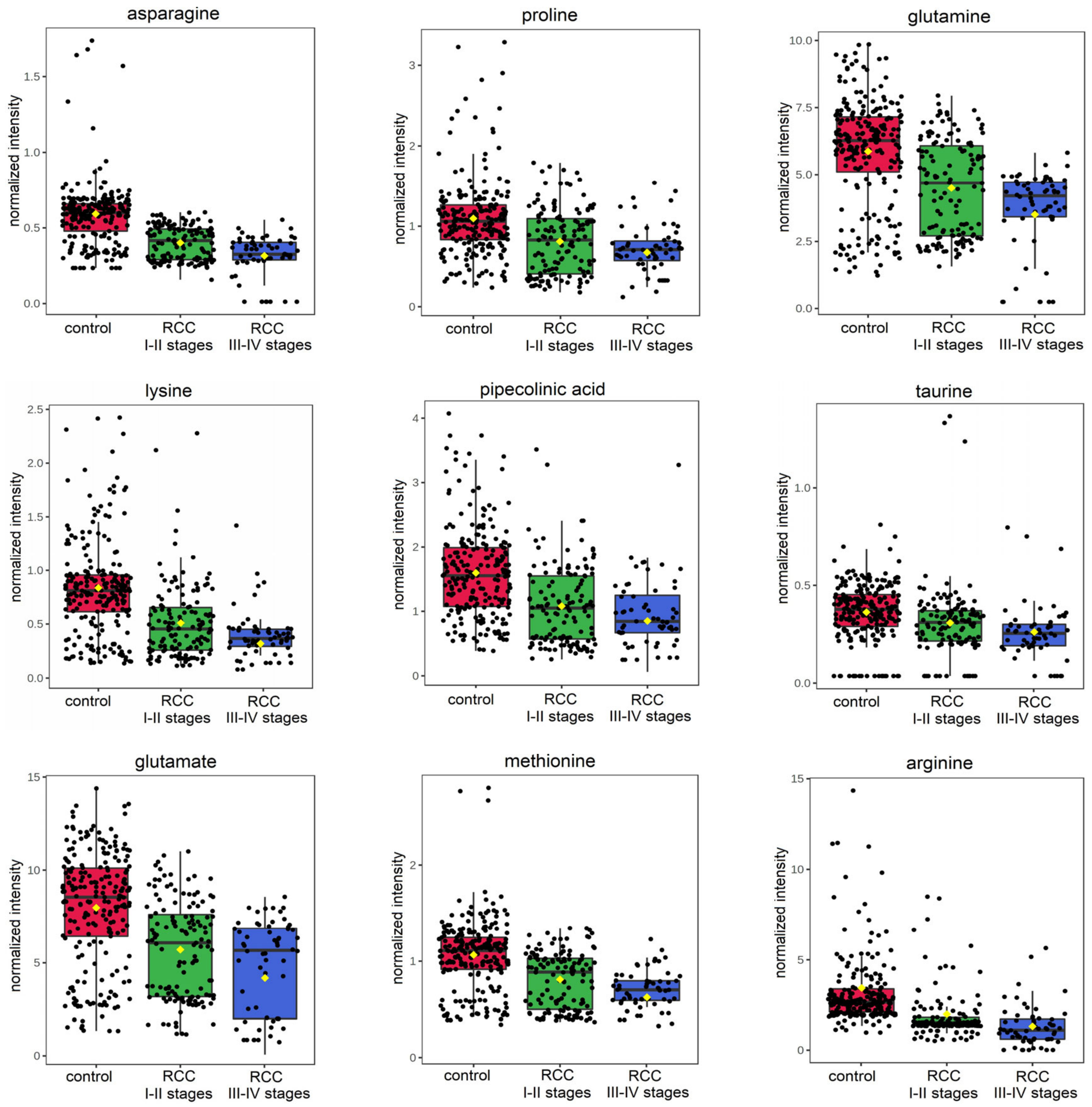

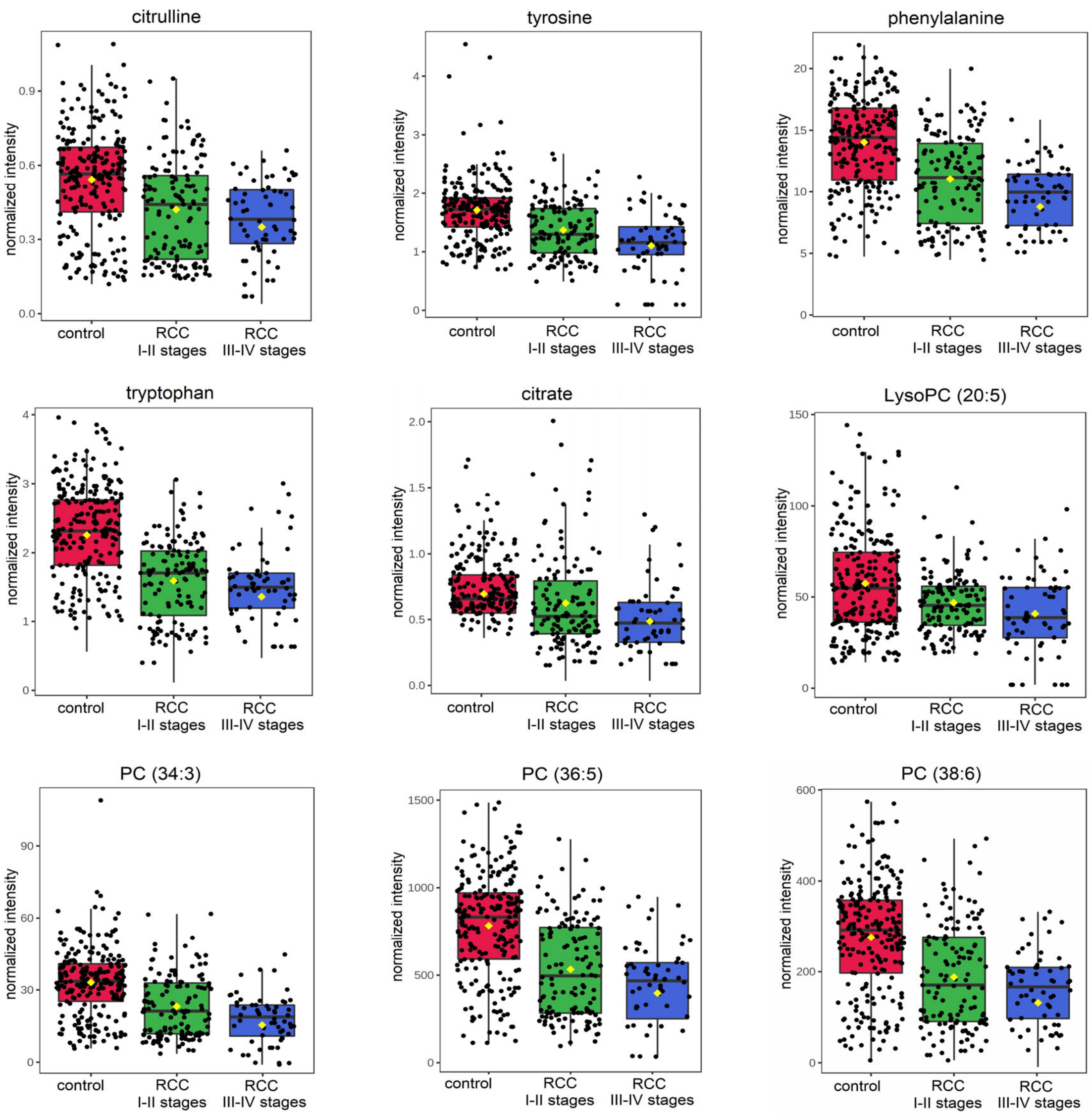

Figure S2. Box and whisker plots of the annotated metabolites between the controls and RCC patients at different stages. The box and whisker plot presents the distribution of normalized intensity values of the annotated metabolites. The top and bottom of the boxes represent the 25% and 75% percentiles; the 5% and 95% percentiles are indicated as error bars. The median value is indicated by horizontal lines within each box.

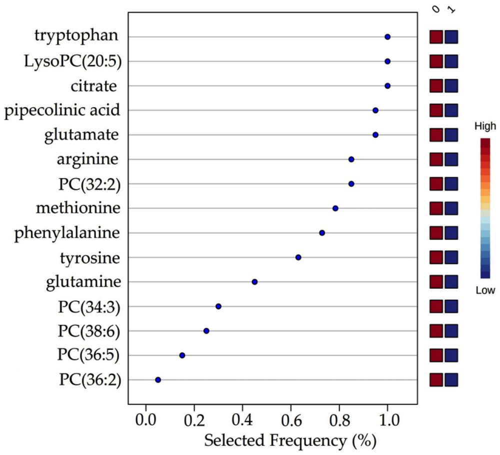

(**a**)

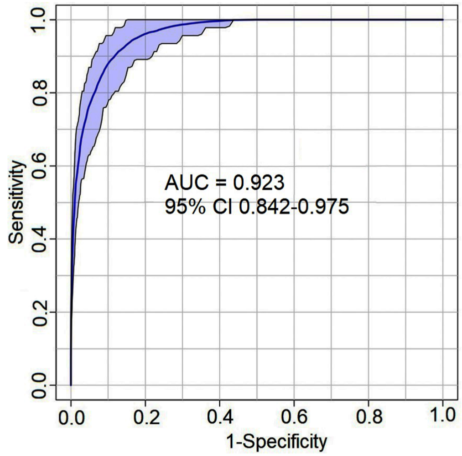

(**b**)

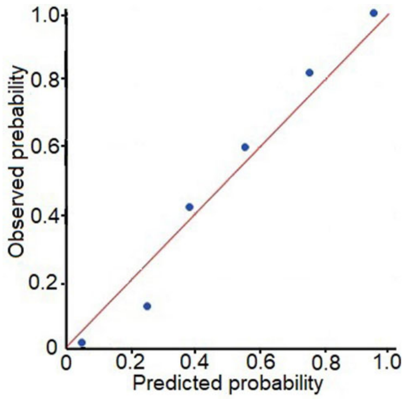

(**c**)

Figure S3. The selection of metabolites for the diagnostic model and its performance evaluation. (**a**) The top frequency metabolites. Random Forest algorithm was used for frequency ranking of the selected metabolites. The colored boxes on the right indicate the intensity of the corresponding metabolite in the groups (0 - control, 1- ccRCC patients). (**b**) The ROC curve of the most optimal diagnostic model (gives the largest AUC and the highest predictive accuracy) that was generated using 10 metabolites. The following mean values were obtained: AUC – 0.92 (95% CI: 0.84-0.98); sensitivity – 0.84 (95% CI: 0.77-0.89); specificity – 0.91 (95% CI: 0.83-0.96). The ROC curve was generated using the results of Monte-Carlo cross validation (MetaboAnalyst 5.0). 95% CI is shown in blue. (**c**) The overfitting-corrected calibration plot. Predicted probability vs. observed probability is shown.

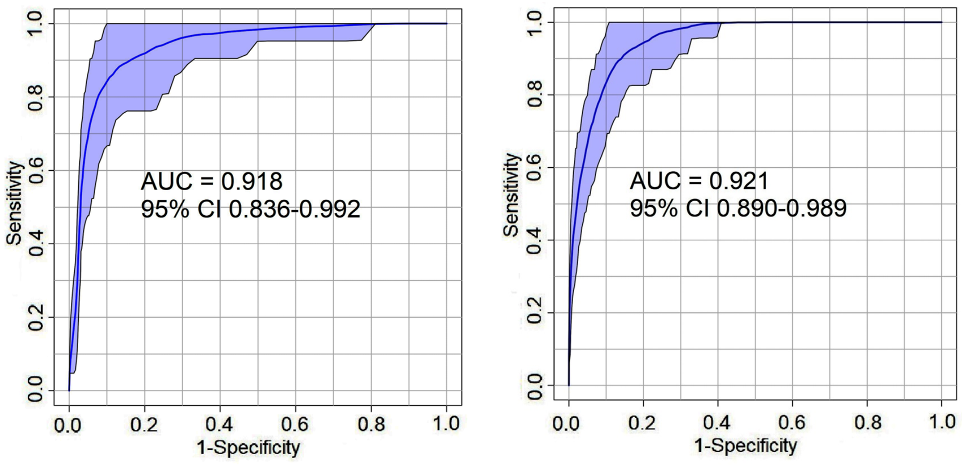

(**a**) (**b**)

Figure S4. The ability of the diagnostic model (generated using 10 metabolites) to discriminate the patients with early stage ccRCC according to their gender. (**a**) The ROC curve was generated using samples of males with early stage ccRCC. The following mean values were obtained: AUC – 0.92 (95% CI: 0.84-0.98); sensitivity – 0.89 (95% CI: 0.81-0.95); specificity – 0.91 (95% CI: 0.80-0.96). (**b**) The ROC curve was generated using samples of females with early stage ccRCC. The following mean values were obtained: AUC – 0.92 (95% CI: 0.89-0.99); sensitivity – 0.86 (95% CI: 0.79-0.92); specificity – 0.97 (95% CI: 0.90-0.99). The ROC curves were generated using the results of Monte-Carlo cross validation (MetaboAnalyst 5.0). 95% CI are shown in blue.

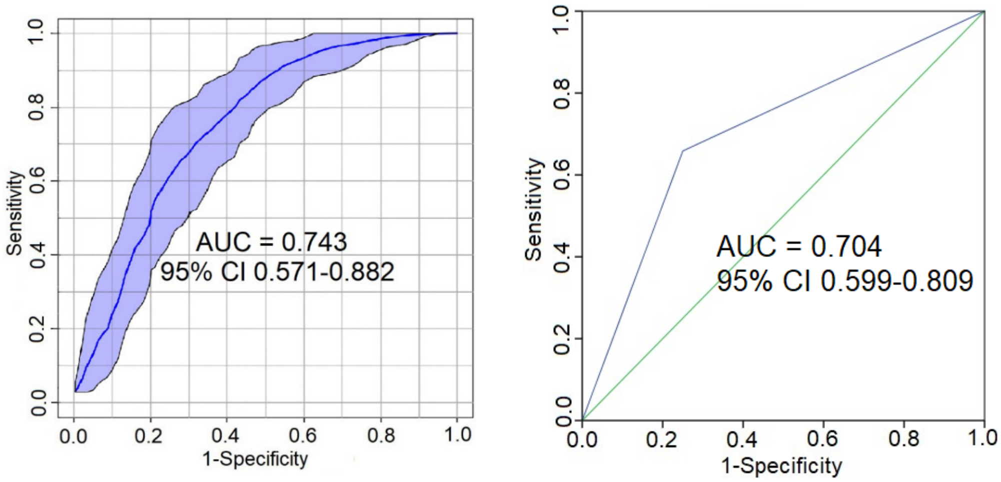

(**a**) (**b**)

Figure S5. Receiver operating characteristic (ROC) curve showing the ability of the diagnostic model (generated using 10 metabolites) to distinguish the early stage ccRCC samples from advanced stage ccRCC samples. (**a**) The ROC curve was generated using the results of Monte-Carlo cross validation (MetaboAnalyst 5.0). The following mean values were obtained: AUC – 0.74 (95% CI: 0.57-0.88); sensitivity – 0.82 (95% CI: 0.75-0.89); specificity – 0.83 (95% CI: 0.73-0.92). 95% CI is shown in blue. (**b**) ROC curve of the diagnostic model obtained on the independent test set. The following values were obtained: AUC–0.70 (95% CI: 0.60–0.81), specificity-0.82 (95% CI: 0.75-0.88), and respectively-0.74 (95% CI: 0.62-0.83). SPSS was used to build the plot.

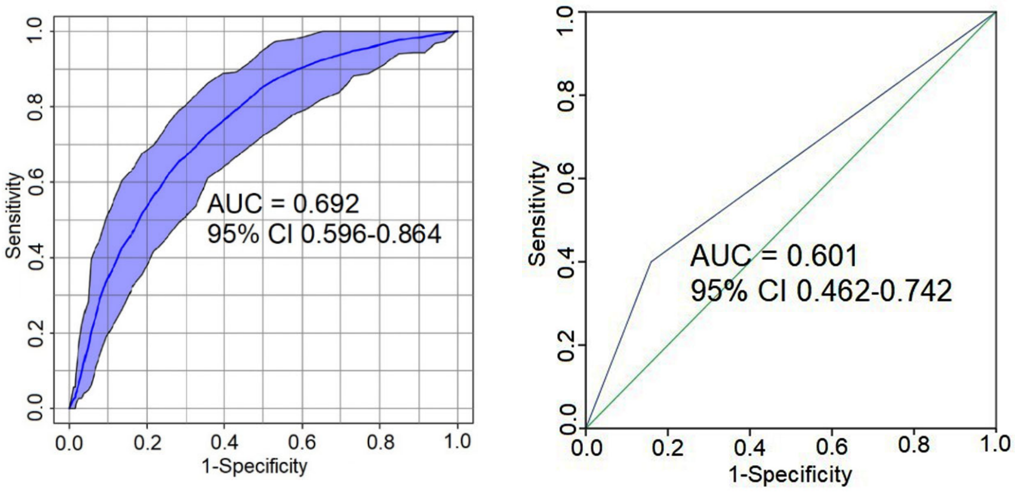

(**a**) (**b**)

Figure S6. Receiver operating characteristic (ROC) curve of the diagnostic model (generated using 10 metabolites) for lung cancer prediction. (**a**) The ROC curve was generated using the results of Monte-Carlo cross validation (MetaboAnalyst 5.0). The following mean values were obtained: AUC – 0.69 (95% CI: 0.60-0.86); sensitivity – 0.78 (95% CI: 0.69-0.85); specificity – 0.75 (95% CI: 0.65-0.81). CI is shown in blue. (**b**) ROC curve of the diagnostic model obtained on the independent test set. The following values were obtained: AUC–0.60 (95% CI 0.46–0.74), sensitivity-0.61 (95% CI: 0.48-0.73), and specificity-0.69 (95% CI: 0.49-0.89). SPSS was used to build the plot.

Table S1. The accuracy results of orthogonal partial least squares discriminant analysis models.

Compared groups	R^2Y	Q^2	p-value
control vs ccRCC patients (III-IV stages)	0.73	0.69	<0.01
control vs ccRCC patients (I-II stages)	0.61	0.52	<0.05
control vs pRCC and chrRCC patients (I-II stages)	0.56	0.50	<0.05

R^2 – coefficient of determination; Q^2 – predictive ability; p-values – validity (evaluated through one hundred permutation validations; p-value ≤ 0.05 is considered as statistically significant).

Table S2. Putatively annotated differential metabolites.

Name of metabolites	Formula	Adduct	m/z	Non-cancer vs ccRCC cancer patients (III-IV stages)		Non-cancer vs ccRCC cancer patients (I-II stages)		Non-cancer vs pRCC/chrRCC cancer patients (I-II stages)	
				P value	VIP value	P value	VIP value	P value	VIP value
threonine	$C_4H_9NO_3$	$[M+H]^+$	104.071	4.24×10^{-3}	0.95	1.95×10^{-4}	1.02	5.34×10^{-3}	1.01
serine	$C_3H_7NO_3$	$[M+H]^+$	106.049	6.02×10^{-4}	1.01	1.05×10^{-4}	0.99	9.36×10^{-3}	0.97
proline	$C_5H_9NO_2$	$[M+H]^+$	116.070	1.11×10^{-4}	1.02	1.95×10^{-3}	0.93	3.23×10^{-3}	0.94
valine	$C_5H_{11}NO_2$	$[M+H]^+$	118.086	5.38×10^{-4}	1.05	2.37×10^{-4}	1.03	4.74×10^{-4}	1.01
delta(1)-piperidine-2-carboxylate	$C_6H_9NO_2$	$[M+H]^+$	128.070	2.12×10^{-8}	1.21	1.11×10^{-4}	1.02	3.93×10^{-4}	1.02
oxoproline	$C_5H_7NO_3$	$[M]^+$	129.042	9.24×10^{-7}	1.21	1.76×10^{-4}	0.97	6.71×10^{-4}	0.95
oxoproline	$C_5H_7NO_3$	$[M+H]^+$	130.049	4.87×10^{-5}	1.27	9.23×10^{-4}	0.99	8.23×10^{-4}	0.97
pipecolinic acid	$C_6H_{11}NO_2$	$[M+H]^+$	130.086	1.37×10^{-10}	1.07	3.11×10^{-8}	1.04	4.01×10^{-7}	1.02
leucine	$C_6H_{13}NO_2$	$[M+H]^+$	132.101	9.69×10^{-3}	1.16	1.42×10^{-5}	1.19	5.92×10^{-4}	1.11
asparagine	$C_4H_8N_2O_3$	$[M+H]^+$	133.061	9.81×10^{-10}	1.04	7.93×10^{-8}	1.08	2.17×10^{-7}	1.07
proline	$C_5H_9NO_2$	$[M+Na]^+$	138.052	9.87×10^{-4}	1.01	2.22×10^{-3}	0.86	6.31×10^{-3}	0.91
valine	$C_5H_{11}NO_2$	$[M+Na]^+$	140.068	8.18×10^{-3}	1.08	2.61×10^{-4}	1.11	4.87×10^{-3}	1.08
3-dehydroxycarnitine	$C_7H_{15}NO_2$	$[M+Na]^+$	146.117	2.43×10^{-6}	1.23	1.31×10^{-2}	1.12	3.53×10^{-2}	1.11
glutamine	$C_5H_{10}N_2O_3$	$[M+H]^+$	147.076	1.22×10^{-8}	1.27	1.11×10^{-9}	1.16	4.21×10^{-8}	1.15
lysine	$C_6H_{14}N_2O_2$	$[M+H]^+$	147.112	9.05×10^{-7}	0.99	7.11×10^{-10}	1.02	7.83×10^{-8}	1.02

taurine	$C_2H_7NO_3S$	$[M+Na]^+$	148.003	6.53×10^{-7}	1.16	4.18×10^{-3}	0.81	3.11×10^{-3}	0.86
glutamate	$C_5H_9NO_4$	$[M+H]^+$	148.060	5.19×10^{-10}	1.19	2.76×10^{-9}	1.15	6.85×10^{-8}	1.14
methionine	$C_5H_{11}NO_2S$	$[M+H]^+$	150.058	1.38×10^{-9}	1.13	2.76×10^{-9}	1.09	3.33×10^{-7}	1.07
leucine	$C_6H_{13}NO_2$	$[M+Na]^+$	154.083	2.54×10^{-4}	1.29	6.69×10^{-6}	1.16	2.05×10^{-6}	1.14
aspartate	$C_4H_7NO_4$	$[M+Na]^+$	156.026	1.23×10^{-3}	1.06	2.83×10^{-6}	1.09	2.83×10^{-5}	1.08
valine	$C_5H_{11}NO_2$	$[M+K]^+$	156.042	1.23×10^{-5}	1.07	2.83×10^{-4}	1.07	3.07×10^{-4}	1.06
histidine	$C_6H_9N_3O_2$	$[M+H]^+$	156.076	6.61×10^{-5}	1.01	6.77×10^{-4}	0.96	6.43×10^{-4}	0.99
L-carnitine	$C_7H_{16}NO_3$	$[M]^+$	162.112	1.20×10^{-6}	1.22	6.10×10^{-5}	1.17	7.13×10^{-4}	1.14
phenylalanine	$C_9H_{11}NO_2$	$[M+H]^+$	166.086	7.17×10^{-5}	1.05	4.20×10^{-3}	1.01	4.72×10^{-3}	1.01
glutamine	$C_5H_{10}N_2O_3$	$[M+Na]^+$	169.058	2.15×10^{-7}	1.31	$1{,}24 \times 10^{-6}$	1.19	1.07×10^{-6}	1.22
asparagine	$C_4H_8N_2O_3$	$[M+K]^+$	171.016	7.77×10^{-3}	1.01	2.93×10^{-3}	0.99	3.63×10^{-3}	0.98
arginine	$C_6H_{14}N_4O_2$	$[M+H]^+$	175.118	2.49×10^{-7}	1.11	2.88×10^{-9}	1.18	5.96×10^{-7}	1.12
citrulline	$C_6H_{13}N_3O_3$	$[M+H]^+$	176.102	2.63×10^{-5}	1.15	1.68×10^{-6}	1.17	9.22×10^{-5}	1.18
tyrosine	$C_9H_{11}NO_3$	$[M+H]^+$	182.081	1.82×10^{-8}	1.18	1.67×10^{-2}	1.02	7.45×10^{-2}	1.01
4-acetamidobutanoate	$C_6H_{11}NO_3$	$[M+K]^+$	184.037	5.63×10^{-9}	1.12	5.57×10^{-4}	1.01	1.04×10^{-4}	1.01
glutamate	$C_5H_9NO_4$	$[M+K]^+$	184.984	3.10×10^{-7}	1.27	3.29×10^{-6}	1.09	5.06×10^{-6}	1.11
glutamine	$C_5H_{10}N_2O_3$	$[M+K]^+$	185.032	1.37×10^{-9}	1.19	1.94×10^{-8}	1.16	3.92×10^{-8}	1.18
hydroxyglutamate semialdehyde	$C_5H_9NO_4$	$[M+K]^+$	186.016	5.32×10^{-7}	1.07	8.23×10^{-4}	1.01	3.72×10^{-4}	1.03
phenylalanine	$C_9H_{11}NO_2$	$[M+Na]^+$	188.068	$1{,}67 \times 10^{-7}$	1.11	$3{,}96 \times 10^{-3}$	0.91	$5{,}68 \times 10^{-3}$	0.88
citrulline	$C_6H_{13}N_3O_3$	$[M+Na]^+$	198.084	1.22×10^{-5}	1.08	5.53×10^{-6}	1.17	8.77×10^{-5}	1.14
tryptophan	$C_{11}H_{12}N_2O_2$	$[M+H]^+$	205.097	9.64×10^{-12}	1.23	2.57×10^{-15}	1.15	7.04×10^{-12}	1.12
citrulline	$C_6H_{13}N_3O_3$	$[M+K]^+$	214.051	1.96×10^{-10}	1.21	$1{,}61 \times 10^{-7}$	1.16	9.04×10^{-6}	1.13

citrate	$C_6H_8O_7$	$[M+Na]^+$	215.016	2.31×10^{-7}	0.97	3.43×10^{-11}	1.22	5.42×10^{-8}	1.09
tryptophan	$C_{11}H_{12}N_2O_2$	$[M+Na]^+$	227.079	7.31×10^{-6}	1.26	6.44×10^{-8}	1.21	6.43×10^{-7}	1.19
acetyl -galactosamine	$C_8H_{15}NO_6$	$[M+Na]^+$	244.078	2.51×10^{-6}	1.03	2.63×10^{-8}	1.06	1.14×10^{-7}	1.08
glucosamine phosphate	$C_6H_{14}NO_8P$	$[M+H]^+$	260.052	4.86×10^{-9}	1.22	3.19×10^{-6}	1.16	7.04×10^{-5}	1.14
C16-sphinganine	$C_{16}H_{35}NO_2$	$[M+H]^+$	274.274	8.88×10^{-3}	1.01	1.16×10^{-3}	1.01	1.01×10^{-3}	1.01
palmitic acid	$C_{16}H_{32}O_2$	$[M+Na]^+$	279.229	1.47×10^{-4}	1.21	1.49×10^{-5}	1.24	7.82×10^{-4}	1.15
C17 sphinganine	$C_{17}H_{37}NO_2$	$[M+H]^+$	288.289	1.63×10^{-3}	1.01	1.09×10^{-3}	1.01	6.85×10^{-3}	1.01
gamma-glutamyltaurine	$C_7H_{14}N_2O_6S$	$[M+K]^+$	293.020	2.33×10^{-5}	1.09	1.64×10^{-2}	0.89	2.54×10^{-2}	0.86
linoleic acid	$C_{18}H_{32}O_2$	$[M+Na]^+$	303.229	9.53×10^{-7}	1.05	3.59×10^{-6}	1.03	7.26×10^{-6}	1.02
oleic acid	$C_{18}H_{34}O_2$	$[M+Na]^+$	305.245	8.39×10^{-4}	1.01	6.40×10^{-4}	1.01	9.12×10^{-3}	1.01
stearic acid	$C_{18}H_{36}O_2$	$[M+Na]^+$	307.261	1.88×10^{-9}	1.12	$2,21\times10^{-8}$	1.11	8.45×10^{-7}	1.08
LysoPC(p16:0)	$C_{24}H_{50}NO_6P$	$[M+H]^+$	480.344	5.79×10^{-4}	1.03	2.73×10^{-4}	1.01	2.72×10^{-3}	1.01
LysoPC(16:0)	$C_{24}H_{50}NO_7P$	$[M+H]^+$	496.339	5.65×10^{-3}	1.04	3.71×10^{-3}	1.03	7.34×10^{-3}	1.04
LysoPC(18:3)	$C_{26}H_{48}NO_7P$	$[M+H]^+$	518.324	3.59×10^{-3}	1.18	2.44×10^{-3}	1.15	3.44×10^{-3}	1.16
LysoPC(18:2)	$C_{26}H_{50}NO_7P$	$[M+H]^+$	520.339	2.70×10^{-9}	1.03	1.29×10^{-7}	1.01	4.75×10^{-7}	1.01
LysoPC(18:1)	$C_{26}H_{52}NO_7P$	$[M+H]^+$	522.355	3.64×10^{-3}	1.01	1.01×10^{-4}	1.01	5.67×10^{-3}	1.01
LysoPC(18:0)	$C_{26}H_{54}NO_7P$	$[M+H]^+$	524.371	1.29×10^{-3}	1.01	1.75×10^{-3}	1.01	6.82×10^{-4}	1.01
LysoPC(20:5)	$C_{28}H_{48}NO_7P$	$[M+H]^+$	542.324	3.98×10^{-12}	1.03	$1,12\times10^{-8}$	1.02	3.98×10^{-6}	1.01
PC(32:2)	$C_{40}H_{76}NO_8P$	$[M+H]^+$	730.538	6.69×10^{-13}	1.02	8.34×10^{-9}	1.01	2.65×10^{-7}	1.01
PC(32:1)	$C_{40}H_{78}NO_8P$	$[M+H]^+$	732.553	8.38×10^{-8}	1.03	7.71×10^{-4}	1.02	6.12×10^{-7}	1.05
PC(34:3)	$C_{42}H_{78}NO_8P$	$[M+H]^+$	756.553	2.74×10^{-10}	1.06	$3,20\times10^{-7}$	1.06	7.22×10^{-6}	1.05
PC(34:2)	$C_{42}H_{80}NO_8P$	$[M+H]^+$	758.569	1.55×10^{-10}	1.04	5.69×10^{-7}	1.01	8.02×10^{-6}	1.01

PC(34:1)	$C_{42}H_{82}NO_8P$	$[M+H]^+$	760.585	1.71×10^{-7}	1.02	5.20×10^{-5}	1.01	7.32×10^{-6}	1.01
PC(36:5)	$C_{44}H_{78}NO_8P$	$[M+H]^+$	780.553	2.37×10^{-12}	1.12	9.58×10^{-8}	1.09	6.69×10^{-7}	1.08
PC(36:3)	$C_{44}H_{82}NO_8P$	$[M+H]^+$	784.585	9.03×10^{-6}	1.05	$2,60 \times 10^{-7}$	1.06	7.04×10^{-6}	1.04
PC(36:2)	$C_{44}H_{84}NO_8P$	$[M+H]^+$	786.601	1.77×10^{-11}	1.02	2.55×10^{-8}	1.01	7.74×10^{-7}	1.01
PC(36:1)	$C_{44}H_{86}NO_8P$	$[M+H]^+$	788.616	2.21×10^{-9}	1.05	1.85×10^{-5}	1.01	8.05×10^{-8}	1.04
PC(38:7)	$C_{46}H_{78}NO_8P$	$[M+H]^+$	804.553	1.22×10^{-6}	1.11	3.61×10^{-7}	1.12	4.92×10^{-6}	1.11
PC(38:6)	$C_{46}H_{80}NO_8P$	$[M+H]^+$	806.569	1.22×10^{-9}	1.14	9.43×10^{-8}	1.18	4.25×10^{-7}	1.12
PC(42:3)	$C_{50}H_{94}NO_8P$	$[M+H]^+$	868.678	7.22×10^{-7}	1.15	4.60×10^{-7}	1.17	5.65×10^{-6}	1.14
PC(42:1)	$C_{50}H_{98}NO_8P$	$[M+H]^+$	872.710	9.95×10^{-6}	1.13	9.70×10^{-7}	1.15	7.23×10^{-6}	1.14

p-value – obtained from pairwise Mann–Whitney *U* test; VIP value – obtained from OPLS-DA

Table S3. Identification of differential metabolites by MS/MS fragmentation.

Precursor Ion					Product Ion			
**Metabolite**	**Registered *m/z*, Da**	**Calculated *m/z*, Da**	**Chemical Formula**	**Ion Type**	**Registered *m/z*, Da**	**Reference *m/z*, Da**	**Chemical Formula**	**Ion Type**
proline	116.0701	116.0706	$C_5H_9NO_2$	$[M + H]^+$	98.0607	98.0600	C_5H_8NO	$[M]^+$
valine	118.0861	118.0862	$C_5H_{11}NO_2$	$[M + H]^+$	102.0549	102.0555	$C_4H_8NO_2$	$[M]^+$
4-oxoproline	130.0497	130.0487	$C_5H_7NO_3$	$[M + H]^+$	84.0444	84.0444	unknown	unknown
leucine	132,1024	132.1019	$C_6H_{13}NO_2$	$[M + H]^+$	86.097	86.0969	$C_5H_{12}N$	$[M]^+$
glutamine	147.0776	147.0764	$C_5H_{10}N_2O_3$	$[M + H]^+$	130.0496	130.056	$C_5H_7NO_3$	$[M + H]^+$
					101.0706	101.07	unknown	unknown
lysine	147.1126	147.1128	$C_6H_{14}N_2O_2$	$[M + H]^+$	130.0860	130.0887	unknown	unknown
					129.1024	129.1022	$C_6H_{13}N_2O$	$[M]^+$
glutamate	148.0603	148.0607	$C_5H_9NO_4$	$[M + H]^+$	130.0496	130.056	$C_5H_7NO_3$	$[M + H]^+$
					102.0550	102.056	$C_4H_8NO_2$	$[M]^+$
					84.0454	84.045	C_4H_6NO	$[M]^+$
methionine	150.0585	150.0583	$C_5H_{11}NO_2S$	$[M + H]^+$	104.0536	104.0529	unknown	unknown
carnitine	162.1126	162.1125	$C_7H_{15}NO_3$	$[M + H]^+$	103.0390	103.039	$C_4H_7O_3$	$[M]^+$
					102.0913	102.0913	$C_5H_{12}NO$	$[M]^+$
					85.0289	85.0284	$C_4H_4O_2$	$[M + H]^+$
phenylalanine	166.0859	166.0862	$C_9H_{11}NO_2$	$[M + H]^+$	147.0304	147.0	unknown	unknown
					131.9872	131.2	unknown	unknown
					120.0806	120.1	unknown	unknown
					102.9499	102.9	unknown	unknown
arginine	175.1242	175.1196	$C_6H_{14}N_4O_2$	$[M + H]^+$	129.9713	129.98	unknown	unknown
					115.9619	115.96	unknown	unknown
tryptophan	205.0926	205.0971	$C_{11}H_{12}N_2O_2$	$[M + H]^+$	170.0618	170.0606	$C_{11}H_8NO$	$[M]^+$
					144.0822	144.0813	$C_{10}H_{10}N$	$[M]^+$
					118.0650	118.0657	C_8H_8N	$[M]^+$

Identification was carried out by matching fragmentation spectra (result of MS/MS fragmentation of differential metabolites) to reference fragmentation spectra of metabolites from the public metabolite databases (HMDB, METLIN); m/z — mass-to-charge ratio. A mass tolerance window— 0.005 Da.

Table S4. A discrimination ability of the model for prediction of early stage ccRCC.

Testing group<br>Patients with early stage ccRCC	AUC	Confidence interval	Sensitivity	Specificity	Accuracy
Male	0.94	0.850-0.992	0.89	0.90	0.89
Female	0.95	0.890-0.989	0.86	0.97	0.91

ROC curves of the models were generated using the results of Monte-Carlo cross validation (MetaboAnalyst 5.0) (100-times repeated 3-fold).
